# A journey through the molecular networks of endomyocardial biopsies to explore ATTR and AL*λ* cardiac amyloidosis

**DOI:** 10.1016/j.isci.2026.116573

**Published:** 2026-07-03

**Authors:** Raffaello Viganò, Andrea Lomagno, Fredrik Noborn, Jonas Nilsson, Saleh Hamed, Anders Oldfors, Kristjan Karason, Kristina Vukusic, Joakim Sandstedt, Emanuele Bobbio, Göran Larson, Pierluigi Mauri, Francesca Brambilla, Dario Di Silvestre

**Affiliations:** 1 Department of Biomedical Science, Institute for Biomedical Technologies - National Research Council (ITB-CNR), 20054 Segrate, Italy; 2 Department of Laboratory Medicine, Institute of Biomedicine, University of Gothenburg, Sahlgrenska University Hospital, 41345 Gothenburg, Sweden; 3 Proteomics Core Facility, Sahlgrenska Academy, University of Gothenburg, 41390 Gothenburg, Sweden; 4 Department of Biomedical Science, Institute of Endotypes in Oncology Metabolism and Immunology “G. Salvatore” - National Research Council (IEOMI-CNR), 80131 Naples, Italy; 5 Department of Clinical Pathology, Sahlgrenska University Hospital, 41345 Gothenburg, Sweden; 6 Department of Cardiology, Sahlgrenska University Hospital, 41345 Gothenburg, Sweden; 7 Department of Transplantation, Sahlgrenska University Hospital, 41345 Gothenburg, Sweden; 8 Department of Clinical Chemistry, Sahlgrenska University Hospital, 41345 Gothenburg, Sweden

**Keywords:** cardiac amyloidosis, endomyocardial biopsy, proteomics, protein-protein nteraction net-work, PPI, protein co-expression network, network topology, network hubs

## Abstract

Immunoglobulin light chains (AL) and transthyretin (ATTR) characterize most cardiac amyloidosis cases. Several proteomic studies have analyzed microdissected amyloid plaques. However, they did not capture the molecular dysfunctions triggered in surrounding tissue. We, therefore, holistically investigated the protein profiles of endomyocardial biopsies from patients with AL*λ* and ATTR conditions. Both conditions showed extracellular matrix remodeling and downregulation of proteostasis, signaling, and mitochondrial energetics. The results also suggested vesicle-mediated transport and complement activation as hallmarks of patients with AL*λ* and ATTR conditions, respectively. These findings corroborate previously proposed pathological mechanisms, namely, direct mitochondrial toxicity of light chain fibrils internalized via macropinocytosis and massive extracellular deposition of ATTR. Notably, protein quantification and network topology enabled the identification of potential disease markers. Although the selection criteria were robust, validation in larger cohorts is needed. Nevertheless, given the challenging accessibility of the analyzed samples, the proposed landscape of results provides a valuable resource to encourage further studies on cardiac amyloidosis.

## Highlights

• Endomyocardial AL*λ* and ATTR biopsies show a similar proteome remodeling
• Vesicle-mediated transport characterizes AL*λ* patients
• Complement activation characterizes ATTR patients
• ADGRL1 and EPPK1 are among the potential markers to be tested in larger cohorts

## Graphical abstract

**Figure:**
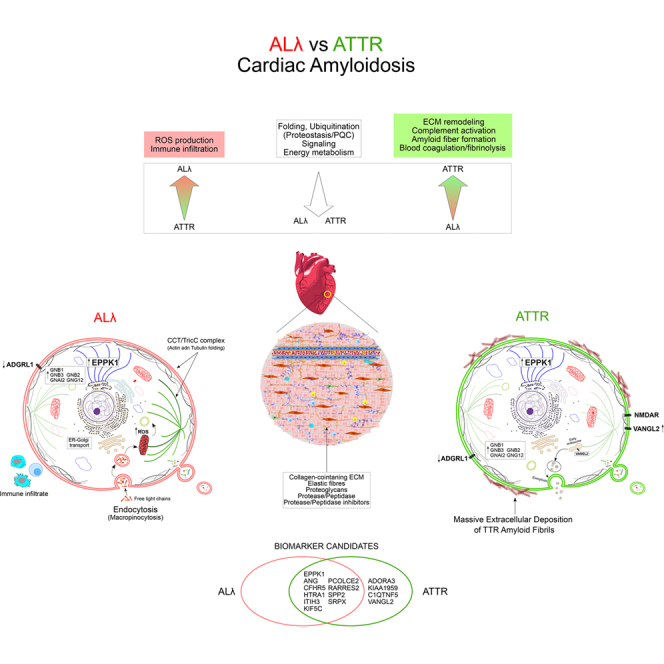

## Teaser

Health sciences

## Introduction

Amyloidosis encompasses a heterogeneous group of acquired or inherited disorders characterized by extracellular deposition of amyloid fibrils in various organs and tissues.^1^ To date, 42 human precursor proteins are known to misfold and self-assemble into amyloid fibrils. Among them, 19 are associated with systemic deposition, while 4 are localized or systemic.^2^

The deposition of protein aggregates in organs and tissues triggers structural and molecular dysfunctions, ultimately leading to functional failure.^3^ However, as seen in immunoglobulin light chains (AL) amyloidosis, direct cytotoxic effects of prefibrillar species are also known.^4^ AL and transthyretin (ATTR) represent the most common phenotypes (≥95%) of cardiac amyloidosis.^5^ ATTR amyloidosis can manifest as a rare hereditary form (ATTRv) or, more frequently, as an age-dependent non-hereditary subtype (ATTRwt). Over recent years, the prevalence of both ATTR and AL cardiac amyloidosis has steadily increased due to improved treatment strategies, leading to better patient survival. Conversely, the incidence of ATTR has risen due to enhanced diagnostic procedures and clinical awareness, whereas that of the AL subtype has remained stable.^6^

Patients with AL and ATTRwt cardiac amyloidosis share similar cardiovascular dysfunctions but often present distinct disease-associated signs.^7^ Macroglossia, periorbital hemorrhages, and albuminuria characterize AL patients, whereas polyneuropathy, gastrointestinal symptoms, and orthopedic pathologies are common in ATTR patients. In ATTRv, specific mutations are associated with particular disease phenotypes.^8^ At the cardiac level, AL patients most frequently display abnormal hemodynamic values, including myocardial stiffness, diastolic and systolic dysfunction, and microvascular obstruction.^9^ Although the latter is more prevalent in ATTR amyloidosis, it has been associated with increased mortality risk only in AL patients. This disparity highlights intrinsic differences in the pathogenesis of ATTR and AL, which may also explain the better prognosis of ATTR, with median survival ranging from 3 to 5 years.^10^ In contrast, due to the direct cardiotoxic effects of circulating free AL, the patients show rapid progression of heart failure symptoms within a few months.^11^

Although organ damage results from the combined effect of amyloid deposition and cytotoxicity of circulating prefibrillar species, these two pathogenic mechanisms carry different weight in ATTR and AL cardiac amyloidosis.^12^ This is reflected in treatments that differ both in action and efficacy.^13^ Indeed, because circulating cytotoxic AL induce rapid cell loss and organ function, recovery occurs only in the early stages of the disease.^14^

Beyond aiming for early diagnosis, understanding the molecular mechanisms of amyloid formation, targeting, and organ damage may pave the way for developing even more effective therapies.^15^ A valuable contribution toward this goal can come from omics analyses of organs and tissues affected by amyloid deposition. Biopsies currently play an important role in achieving an accurate diagnosis and defining disease etiology through in-depth characterization of amyloid plaques.^16^ This information has also been correlated with a patient’s distinctive clinical signs.^17^ Nevertheless, analysis of amyloid plaques alone is insufficient to provide insights into the molecular and histological alterations induced in resident cells of organs and tissues affected by amyloid deposition.

Whole-tissue proteomic analysis may elucidate biological processes and pathways dysregulated by amyloid deposition, but only a limited number of such studies have been conducted to date. In this context, Cai et al. analyzed proteins from primary amyloidosis skin lesions and normal skin tissues by combining iTRAQ, Kyoto Encyclopedia of Genes and Genomes (KEGG), and protein-protein interaction (PPI) analyses.^18^ We recently investigated protein changes in abdominal subcutaneous adipose tissue of patients affected by AL*λ* and AL*κ* amyloidosis using proteomics-data-driven systems biology approaches.^19^ Moreover, only recently, Netzel et al. performed a proteomic analysis of endomyocardial biopsies from ATTR and AL patients.^20^

To expand knowledge of the molecular mechanisms characterizing the hearts of cardiac amyloidosis patients, we investigated here the protein profiles of whole endomyocardial biopsies collected from controls and patients, analyzed retrospectively.^21^ In addition to deepening our understanding of the molecular mechanisms induced by amyloid deposition and the associated organ damage, we aimed to identify the molecular hallmarks of AL*λ* and ATTR cardiac amyloidosis. For these purposes, we adopted a holistic strategy based on PPI and protein co-expression network models.^22^ Two different subject cohorts were selected. The first cohort included equal numbers of control participants and patients affected by AL*λ* and ATTR amyloidosis (*n* = 12 per group), while the second cohort was designed to investigate proteomic differences between AL*λ* (*n* = 19) and ATTR (*n* = 20) endomyocardial biopsies.

## Results

### Subject cohorts, biometric data, and protein profiling

**Figure 1:**
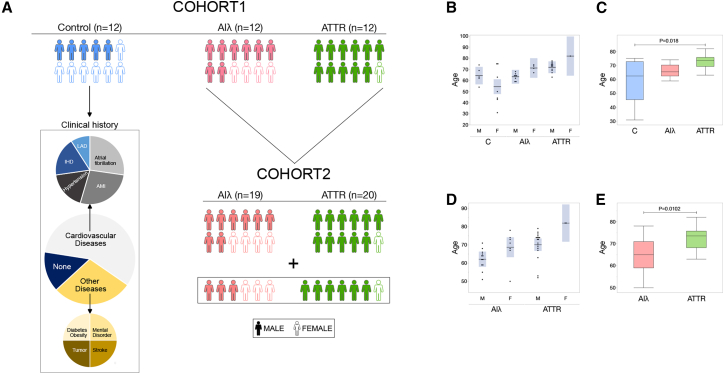
Subject cohorts under investigation (A) Cohort 1 and cohort 2 gender distribution. The clinical history of the control group is reported through pie charts. (B and C) Cohort 1 age distribution, by gender and overall (Tukey-Kramer test, *p* ≤ 0.05; *n* = 12 per group); data are represented as a combination of mean, quantiles (25%–75%), and outliers. (D and E) Cohort 2 age distribution, by gender and overall (Student’s *t* test, *p* ≤ 0.05; AL*λ*, *n* = 19; ATTR, *n* = 20); data are represented as a combination of mean, quantiles (25%–75%), and outliers.

To pursue the study objectives, we established two different subject cohorts, hereinafter referred to as cohort 1 and cohort 2 (Table S1). Cohort1 comprised an equal number of control participants and AL*λ* and ATTR patients (*n* = 12 per group). Differently, cohort2, including a larger number of patients (AL*λ*, *n* = 19 and ATTR, *n* = 20), was designed to shed light on the differences between AL*λ* and ATTR (Figure 1).

Control endomyocardial biopsies in cohort 1 were collected from the hearts of 5 men and 7 women aged between 31 to 75 years. Most participants had a clinical history characterized by various cardiovascular pathologies, predominantly atrial fibrillation and acute myocardial infarction (AMI) (Figure 1A). Cryostat sections of left ventricular sample were stained with hematoxylin and eosin (H&E) to verify representativeness and normal histology and with Congo red to exclude amyloid deposition.^21^ The AL*λ* group included 8 men and 4 women aged between 76 to 98 years, whereas the ATTR group exhibited a higher prevalence of male participants aged between 80 to 96 years. On average, ATTR patients were significantly older than both control participants and AL*λ* patients. Based on ATTR amyloidosis incidence, diagnosis timing, and life expectancy, this trend was anticipated^12^^,^^23^^,^^24^^,^^25^ and similarly characterized cohort2. Moreover, most ATTR patients (90%) had non-hereditary ATTR amyloidosis (ATTRwt) (Table S1).

Proteomic analysis of cohort 1 endomyocardial biopsies enabled global identification of 9,255 distinct proteins (Figures S1A–S1C; Table S2). Over nine thousand distinct proteins were also identified in cohort 2 (Figures S1D–S1F; Table S2). Both AL*λ* and ATTR groups displayed good intra-group homogeneity (Figure S1G). Notably, despite apparent heterogeneity, control participants showed the highest intra-group correlation score (0.933), whereas, as expected, the highest inter-group correlation emerged while comparing AL*λ* and ATTR patients (0.970).

These observations provide a solid foundation for further analyses. However, because age and gender can influence cardiac proteomics, statistical adjustments were applied to quantitative analyses. In this context, prior to inter-groups quantitative comparison, we assessed within each group whether and to what extent age and gender influenced protein abundance (Table S3). Among all the comparisons performed, only two proteins were found to be differentially abundant, both within the control group. Specifically, the eukaryotic translation initiation factor 1A, Y-linked (EIF1AY), whose gene is located on the Y chromosome,^26^ was more abundant in males. Conversely, NLR family pyrin domain containing 11 (NLRP11), a component of the NLRP3 inflammasome known to be activated with aging,^27^ was more abundant in older control participants aged between 60 to 75 years compared with those aged between 30 to 50 years.

### AL*λ* and ATTR patients from cohort 1 exhibit extensive overlap in their endomyocardial proteome

**Figure 2:**
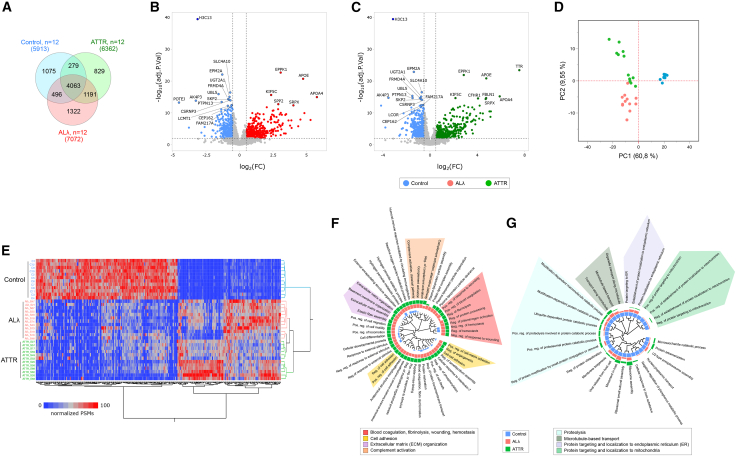
Cohort 1 proteome remodulation (A) Venn diagram of protein identified in control, AL*λ*, and ATTR (*n* = 12 per group). (B) Volcano plot of DAPs in AL*λ* (adj.P.Val ≤0.00001, −0.5 ≥log_2_(FC) ≥0.5). (C) Volcano plot of DAPs in ATTR (adj.P.Val ≤0.00001, −0.5 ≥log_2_(FC) ≥0.5). (D) Principal-component analysis performed using high-confidence DAPs (adj.P.Val ≤0.00001); PC1 explains 60.8% of the variance while PC2 explains 9.55%. (E) Hierarchical clustering (Ward method and Euclidean distance metric) performed using high-confidence DAPs (adj.P.Val ≤0.00001). (F) Biological processes (BPs) most enriched in AL*λ* and ATTR patients. (G) BPs most enriched in control group. Quantitative analysis was performed using limma R package; linear model was fitted to log2-transformed data, including age and gender as covariates. Functional enrichment analysis considered each subject, first individually and then aggregated into the corresponding group. Scale bars in circular layout graphs indicate the normalized number of proteins found per BP and per condition. Hierarchical clustering trees group BPs based on the number of proteins they share. BPs were enriched by FDR ≤0.05, while those differentially enriched were extracted by Linear Discriminant Analysis (LDA) (*p* ≤ 0.01) and Differential Average index (DAve) index ≥|0.4| (see Table S5).

Following proteomic analysis of cardiac biopsies from cohort 1, 4,063 proteins were detected in at least one participant per group, while approximately 1,000 per group were uniquely identified (Figure 2A). Compared with controls, 1,045 (529 upregulated and 516 downregulated) and 1,133 (438 upregulated and 695 downregulated) proteins showed statistically significant differences in AL*λ* and ATTR patients, respectively (Figures 2B and 2C; Table S4). Hierarchical clustering revealed that the proteome of both amyloidosis groups differed markedly from that of controls, with samples clustering according to disease subtype (Figures 2D and 2E). Notably, both principal-component analysis (PCA) and hierarchical clustering heatmap reaffirmed an excellent homogeneity of the control group, consistent with the intra-group correlation reported earlier (Figure S1G).

The protein profiles of both AL*λ* and ATTR were enriched with proteins involved in extracellular matrix (ECM) organization, cell adhesion, complement activation and regulation of blood coagulation, fibrinolysis, wound healing, and hemostasis (Figure 2A and Table S5). Conversely, proteins implicated in intracellular processes were underrepresented. These included proteolysis (especially positive regulation terms, i.e., activation), microtubule-based transport, and protein targeting and localization, particularly to the endoplasmic reticulum (ER) and mitochondria (Figure 2B). Consistent with the aforementioned biological processes, the most enriched molecular functions included proteoglycan binding, glycosaminoglycan binding, and endopeptidase inhibitory activity (Figure S2A). Conversely, both patient groups were low-enriched for proteins located in the mitochondrial ribosome, spindle microtubule, and postsynaptic cytosol (Figure S2B; Table S5).

The functional enrichment analysis findings were corroborated by a more detailed holistic evaluation of PPI network models. To this end, the most significant Differentially Abundant Proteins (DAPs) (adj.P.Val ≤0.00001) were used to construct a PPI network model, with nodes grouped according to their functional and topological properties. Alongside ECM-related proteins, those involved in amyloid fiber formation were over-accumulated in both AL*λ* and ATTR (Figure S3). In contrast, most functional modules were downregulated, including intracellular proteins involved in signaling, mitochondrial energetics, and cardiac muscle processes. Furthermore, ubiquitination and protein folding were downregulated, paralleled by an increase in the expression of protease/peptidase inhibitors. These processes are part of proteostasis and protein quality control (PQC) (autophagy, unfolded protein response [UPR], and ubiquitin-proteasome system), which are critical for cellular homeostasis; their impairment is considered relevant to the etiology of misfolding diseases.^28^

### A set of distinct protein signatures characterizes the endomyocardial proteome of AL*λ* and ATTR patients from cohort 2

**Figure 3:**
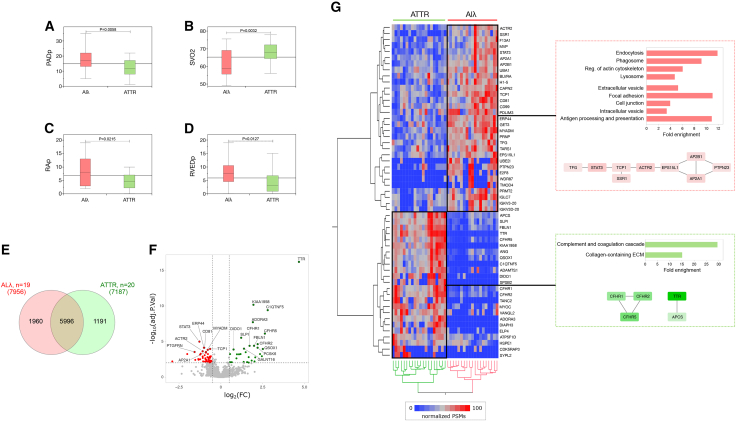
Cohort 2: AL*λ* vs. ATTR comparison (A) Pulmonary artery diastolic pressure (PADp, mmHg). (B) Mixed venous oxygen saturation (SVO2, %). (C) Right atrial pressure (RAp, mmHg). (D) Right ventricular end-diastolic pressure (RVEDp, mmHg). PADp, SVO2, RAp, and RVEDp differences were evaluated by Student’s *t* test; data are represented as a combination of mean, quantiles (25%–75%), and outliers. (E) Venn diagram of protein identified in AL*λ* and ATTR. (F) Volcano plot of DAPs in AL*λ* and ATTR (adj.P.Val ≤0.05, −0.5 ≥log_2_(FC) ≥0.5). Quantitative analysis was performed using limma R package; linear model was fitted to log2-transformed data, including age and gender as covariates. (G) Hierarchical clustering (Ward method and Euclidean distance metric; *n* DEPs = 55, adj.P.Val ≤0.05). Pink and red boxes include biological processes enriched from proteins most abundant in AL*λ* vs. ATTR, respectively (FDR ≤0.5); the most connected PPI components are shown.

Cohort 2 was designed to identify protein signatures distinguishing the AL*λ* and ATTR patients under investigation. In this cohort, AL*λ* patients exhibited higher right atrial pressure (RAp) and pulmonary artery diastolic pressure (PADp). Consistent with more advanced heart failure, they also showed higher right ventricular end-diastolic pressure (RVEDp) and lower mixed venous oxygen saturation (SVO2) (Figures 3A–3D). Meanwhile, no differences were observed for other hemodynamic measurements (Table S1). At the molecular level, comparative proteomic analysis identified 5,996 shared proteins, while 1,960 and 1,191 proteins were found exclusively in AL*λ* and ATTR, respectively, prefiguring protein signatures associated with amyloidosis subtype (Figure 3E).

Preliminary analysis of AL*λ* and ATTR patients included in cohort 1 allowed the identification of 191 DAPs (Figure S4). The larger patient number in cohort 2 was aimed to extracting a more robust and representative set of candidate protein signatures. Following quantitative comparison, 55 proteins showed statistically significant differences (Figures 3F; Table S4). Functionally, proteins more abundant in ATTR were enriched in the complement and coagulation cascade and in collagen-containing ECM. In contrast, those more abundant in AL*λ* were involved in the regulation of the actin cytoskeleton, vesicle-mediated transport, and immune system (Figures 3G; Table S6). In combination with the expression that AL*λ*-specific proteins show in immune cells (Figure S5), these findings support the hypothesis that immune cells infiltrate cardiac tissue following amyloid deposition,^29^ and that this phenomenon is more pronounced in AL*λ*.^23^

To identify candidate disease subtype markers, we selected a set of proteins based on their identification frequency (IF) (Table 1). A first group was identified in all control participants and in no amyloidosis patient in both cohort 1 and cohort 2. Some of these proteins are involved in proteolysis (FAU and SKP2), microtubule dynamics (MTCL2 and CEP162), and Golgi-ER transport (SRP19 and MTCL2), corroborating the earlier global enrichment analysis (Figures 2H–2G). A second group was identified in all amyloidosis patients considered and never in controls. In addition to ECM organization (FBN2 and PCOLCE2) and ECM proteolysis (HTRA1, ITIH3, and SPP2), this group includes pro-inflammatory (RARRES2) and mechanical stress response (EPPK1) proteins. Notably, three proteins, complement C1q tumor necrosis factor-related protein 5 (C1QTNF5), adenosine receptor A3 (ADORA3), and uncharacterized protein KIAA1958 (KIAA1958), were identified in all ATTR patients (and never in controls or AL*λ* patients). In contrast, this did not happen for AL*λ*.

**Table 1:** Differentially abundant proteins specifically identified in control participants or AL*λ* and ATTR patients

	Cohort 1^a^									Cohort 2^b^				
	IF (%)			av.PSMs			adj.P.Val			IF (%)		av.PSMs		adj.P.Val
Protein name	C	AL*λ*	ATTR	C	AL*λ*	ATTR	C vs. AL*λ*	C vs. ATTR	AL vs. ATTR	AL*λ*	ATTR	AL*λ*	ATTR	AL vs. ATTR
Centrosomal protein of 162 kDa (CEP162)	100	0	0	0.52	0	0	4.3E^−13^	3.9E^−13^	–	0	0	0	0	–
CCR4-NOT transcription complex subunit 3 (CNOT3)	100	0	0	0.5	0	0	3.4E^−8^	3.1E^−8^	–	0	0	0	0	–
Cysteine/serine-rich nuclear protein 3 (CSRNP3)	100	0	0	0.61	0	0	1.2E^−14^	7.8E^−15^	–	0	0	0	0	–
Ubiquitin-like FUBI-ribosomal protein eS30 fusion protein (FAU)	100	0	0	0.46	0	0	8E^−8^	7.5E^−8^	–	0	0	0	0	–
N-lysine methyltransferase (KMT5A)	100	0	0	0.48	0	0	7.4E^−9^	6.6E^−9^	–	0	0	0	0	–
Leukotriene B4 receptor 2 (LTB4R2)	100	0	0	0.54	0	0	3E^−11^	2.3E^−11^	–	0	0	0	0	–
Nucleosome assembly protein 1-like 5 (NAP1L5)	100	0	0	0.37	0	0	1.2E^−10^	9.5E^−11^	–	0	0	0	0	–
S-phase kinase-associated protein 2 (SKP2)	100	0	0	0.7	0	0	7.9E^−15^	5.1E^−15^	–	0	0	0	0	–
Microtubule cross-linking factor 2 (SOGA1)	100	0	0	0.37	0	0	1.5E^−10^	1.2E^−10^	–	0	0	0	0	–
Signal recognition particle 19 kDa protein (SRP19)	100	0	0	0.39	0	0	3.9E^−9^	3.3E^−9^	–	0	0	0	0	–
Sulfotransferase 2B1 (SULT2B1)	100	0	0	0.48	0	0	3.6E^−12^	2.7E^−12^	–	0	0	0	0	–
Zinc finger protein 233 (ZNF233)	100	0	0	0.43	0	0	7.8E^−12^	6.2E^−12^	–	0	0	0	0	–
Zinc finger protein 700 (ZNF700)	100	0	0	0.42	0	0	1.1E^−9^	9.2E^−10^	–	0	0	0	0	–
Angiogenin (ANG)	0	100	100	0	2.9	9.7	1.3E^−6^	1.6E^−12^	3.2E^−4^	95	100	2.2	9.3	4.8E^−3^
Complement factor H-related protein 5 (CFHR5)	0	100	100	0	2.8	20.6	2E^−4^	5.3E^−13^	7.7E^−7^	95	100	2.1	19	3.3E^−5^
Epiplakin (EPPK1)	0	100	100	0	7.5	6.4	2.1E^−23^	1.2E^−22^	–	100	100	7.1	5.7	–
Fibrillin-2 (FMN2)	0	100	100	0	5	3.6	2.1E^−11^	1.4E^−9^	–	100	100	3.9	3.1	–
Serine protease (HTRA1)	0	100	100	0	7.6	10.8	2E^−8^	2.5E^−9^	–	95	100	5.7	8.2	–
Inter-alpha-trypsin inhibitor heavy chain H3 (ITIH3)	0	100	100	0	8.7	5.9	5.5E^−7^	4.6E^−5^	–	95	100	6.4	5	–
Kinesin heavy chain isoform 5C (KIF5C)	0	100	100	0	4.3	3.6	2.3E^−16^	2.2E^−15^	–	100	100	3.9	3.5	–
Procollagen C-endopeptidase enhancer 2 (PCOLCE2)	0	100	100	0	12.9	28.3	2E^−7^	1.5E^−10^	–	95	100	10.3	24	–
Retinoic acid receptor responder protein 2 (RARRES2)	0	100	100	0	2.6	3.7	6.2E^−4^	7.2E^−5^	–	89	100	1.9	3.6	–
Secreted phosphoprotein 24 (SPP2)	0	100	100	0	7.1	2.8	1.8E^−13^	1E^−8^	1.3E^−3^	100	100	5.2	2.9	–
Sushi repeat-containing protein (SRPX)	0	100	100	0	19.1	27.6	4.3E^−13^	4.3E^−15^	–	89	95	2.7	5.4	–
SPRY domain-containing SOCS box protein 2 (SPSB2)	0	0	58	0	0	0.72	–	8.2E^−4^	7.9E^−3^	0	65	0	0.61	1E^−02^
Protein diaphanous homolog 3 (DIAPH3)	0	0	75	0	0	1.18	–	4.1E^−5^	6.2E^−4^	0	80	0	1.12	2.8E^−2^
Complement C1q tumor necrosis factor-related protein 5 (C1QTNF5)	0	0	100	0	0	7.93	–	2.5E^−11^	3.9E^−10^	0	95	0	6.07	1.4E^−5^
Adenosine receptor A3 (ADORA3)	0	0	100	0	0	3.45	–	9.1E^−10^	9.1E^−10^	0	100	0	3.23	6.2E^−7^
Uncharacterized protein (KIAA1958)	0	0	100	0	0	3.23	–	4E^−12^	6.8E^−11^	0	100	0	2.98	6.1E^−10^

For each protein, the identification frequency (IF), the average peptide spectrum matches (av.PSMs), and the adjusted *p* value (adj.P.Val) per pairwise comparison are shown for both cohort 1 and cohort 2.

a Control, *n* = 12; AL*λ*, *n* = 12; ATTR, *n* = 12.

b AL*λ*, *n* = 19; ATTR, *n* = 20.

### Unweighted and weighted network topologies reveal details in the endomyocardial proteomic landscape of AL and ATTR patients

Network topology was employed to identify candidate key proteins from a perspective distinct from classical quantitative analysis. Furthermore, integrating these two approaches aims to select more robust candidates by leveraging their complementary strengths.

**Figure 4:**
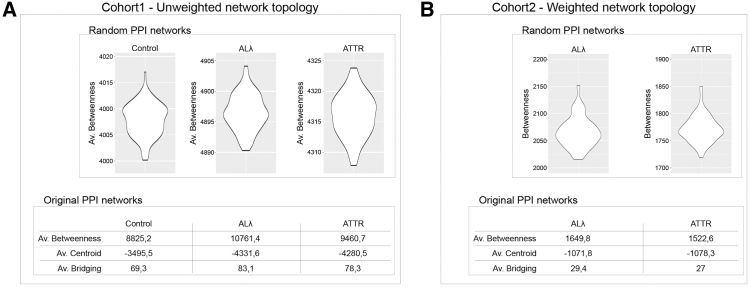
Hubs/bottlenecks validation (A) Unweighted network topology. (B) Weighted network topology. Cohort1 unweighted PPI networks: control (5,684 nodes, 233,300 edges), AL*λ* (6,802 nodes, 279,547 edges), and ATTR (6,102 nodes, 243,658 edges); cohort 2 weighted PPI networks: AL*λ* (1,170 nodes and 26,191 edges) and ATTR (1,170 nodes and 26,191 edges). The statistical significance of the model’s robustness was assessed by comparing the average unweighted and weighted betweenness of the original networks with the betweenness distribution (violin plots) of the corresponding random networks (*n* = 1,000 per condition). These values consistently differ, indicating a shift away from random behavior and supporting the hubs/bottlenecks selection.

Cohort 1 and cohort 2 were analyzed using unweighted and weighted PPI networks, respectively (Table S7). Proteins exhibiting hub and bottleneck properties were validated through random network models, thus confirming that their mean betweenness values differed significantly from those observed in the original PPI networks (Figure 4).

### Unweighted hubs/bottlenecks

**Figure 5:**
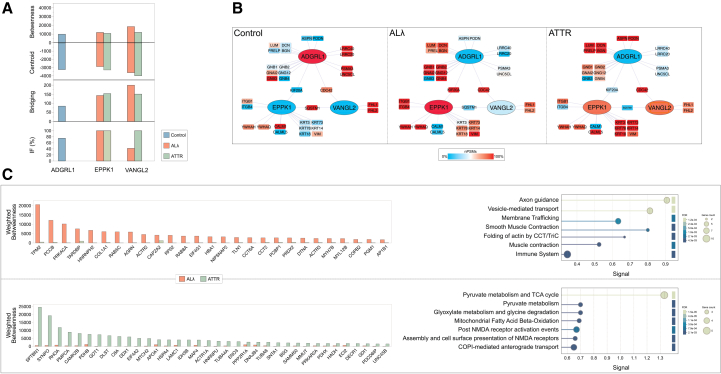
Unweighted and weighted network topological analysis (A) Most significant unweighted hubs/bottlenecks specifically identified in AL*λ* and ATTR patients or control participants, included in cohort 1; identification frequency (IF, *n* = 12), betweenness, centroid, and bridging centrality values are shown. (B) Protein-protein interaction (PPI) subnetworks, including ADGRL1, APPK1, and VANGL2 first neighbors, and the abundance level in control, AL*λ*, and ATTR, respectively. (C) Most significant weighted hubs/bottlenecks in AL*λ* (red) and ATTR (green) extracted by CoPPIs algorithm. The boxes on the right show the corresponding enriched reactome pathways (FDR≤0.05). Betweenness, bridging, and centroid centrality were calculated. Hubs/bottlenecks were extracted by considering the proteins whose centrality values were above the 75th quantile in AL*λ* (or ATTR), but not in the 75th quantile of ATTR (or AL*λ*).

From the set of topologically relevant proteins, the most representative ones were selected based on their frequency of identification (Figure 5A; Table S7). For instance, adhesion G protein-coupled receptor L1 (ADGRL1) was the top hub/bottleneck of control group, where it was identified in 75% of participants, but never in AL*λ* or ATTR patients. Conversely, VANGL planar cell polarity protein 2 (VANGL2) and epiplakin 1 (EPPK1) were never identified in controls. Noteworthy is that EPPK1 was identified in 100% of all patients examined, in both cohort 1 and cohort 2 (Table 1). EPPK1 is a cytoskeletal linker protein that connects intermediate filaments and governs their reorganization in response to mechanical stress.^30^ Its expression showed the same trend as that of its interactors belonging to keratin family (Figure 5B). In particular, keratin 18 (KRT18) and keratin 8 (KRT8) were previously described as cardioprotective, maintaining normal intercalated discs structure as well as mitochondrial integrity and functionality.^31^ Similarly, VANGL2, which regulates tissue polarity and mechanosignaling,^32^ has been implicated in myocardial remodeling from morphological changes to mitochondria via Wnt/JNK signaling.^33^ On the other hand, the abundance of ADGRL1 and some of its interactors was inversely correlated. In addition to collagen-containing ECM proteins (LUM, DCN, PRELP, and BGN), these interactors included several heterotrimeric guanine nucleotide-binding regulatory proteins, namely G protein subunit beta 1 (GNB1), G protein subunit beta 2 (GNB2), G protein subunit beta 4 (GNB4), G protein subunit alpha I2 (GNAI2), and G protein subunit gamma 12 (GNG12). These regulate various biochemical functions through G protein-coupled receptors (GPCRs) located on the plasma membrane.^34^

### Weighted hubs/bottlenecks and differentially correlated functional modules

Thanks to the larger number of patients in cohort 2, we could reconstruct and analyze weighted PPI network models and extract differentially correlated functional modules in addition to weighted hubs/bottlenecks.^22^ AL*λ* weighted hubs/bottlenecks were enriched in proteins involved in vesicle-mediated transport. Additionally, enrichment emerged for axon guidance, immune system, muscle contraction, and folding by CCT/TricC complex. Conversely, ATTR hubs/bottlenecks were enriched in proteins primarily involved in synaptic and mitochondrial-related processes, including pyruvate metabolism, the TCA cycle, mitochondrial fatty acid beta-oxidation, and NMDA receptors (Figure 5C and Table S7). However, in ATTR, we observed a significant correlation between apolipoprotein A1 (APOA1), apolipoprotein A2 (APOA2), and apolipoprotein C3 (APOC3) (Table S8 and Figure S6). Their correlation may indicate the modulation of the pathways involved in regulating cholesterol and triglyceride levels, potentially linked to dyslipidemia.^35^ Indeed, APOA1 and APOA2 participate in reverse cholesterol transport, exerting beneficial effects on cardiovascular health, whereas APOC3 is a lipoprotein lipase inhibitor considered proatherogenic.^36^ In this context, the ATTR group was characterized by correlation among HADHA, HADHB, and ACADVL, such as key enzymes of very long-chain fatty acid beta-oxidation located in the inner mitochondrial membrane. Differently, other beta-oxidation-related proteins, including peroxisome-localized enzymes for short/branched chain degradation, were more correlated in the AL*λ* group.

Other groups of proteins strongly correlated in the ATTR (vs. AL*λ*) group caught our attention. These included coagulation factors (F8, F9, and F10) and the Na^+^/K^+^ transport alpha subunits of the ATPase complex (ATP1A1, ATP1A2, and ATP1A3). The coagulation factors may be associated with thromboembolic events and an increased bleeding risk, which are among the most typical non-cardiac complications of patients with ATTR.^37^^,^^38^ ATP1A1, ATP1A2, and ATP1A3 belong to an enzyme complex embedded in the sarcolemmal membrane, where they transport three Na^+^ and two K^+^, generating a transmembrane gradient essential for maintaining cation homeostasis and regulating contractile force. Their relationship with heart failure is unclear; several studies have reported conflicting evidence of increased, unchanged, and reduced Na^+^/K^+^ ATPase activities under both experimental and clinical conditions.^39^ However, the stronger correlation in the ATTR group could mean better subunit stoichiometric regulation, hence better functioning compared to the AL*λ* group.^40^

## Discussion

In this study, we investigated the protein profiles of endomyocardial biopsies from AL*λ* and ATTR cardiac amyloidosis patients.^21^ They were organized in two different cohorts to highlight similarities and differences. Unlike a previous similar research,^20^ we also used a control group. Furthermore, protein profiles were transformed to molecular networks and analyzed holistically in order to identify impaired biological processes and critical target candidates, i.e., putative biomarkers and hubs/bottlenecks nodes.^22^

Overall, the landscape depicted corroborates hypotheses about the pathogenic mechanisms underlying these phenotypes, such as direct mitochondrial toxicity of AL fibrils, and massive extracellular accumulation of ATTR fibrils that compromise myocardium biomechanic.^12^^,^^23^ On the other hand, both groups of patients considered in the study shared much of the proteomic remodeling. In this scenario, most intracellular functional modules were downregulated. They included signaling, mitochondrial energetics, and cardiac muscle processes, which correlate with impaired cardiac function.^41^ Other evidences suggested alteration of proteolysis, microtubule-based transport, and protein targeting to the ER and mitochondria. Since these processes are part of proteostasis and PQC, including the UPR,^42^ they could also have a link with disease etiology. In this regard, UPR inactivation was recently correlated with systemic amyloid A deposition in mice.^43^ Similarly, stress-independent activation of UPR transcription factors attenuated the secretion of amyloidogenic immunoglobulin AL, reducing their proteotoxicity.^44^

It should be considered that the impairment of proteostasis and PQC could be the consequence of a mitochondrial energetic alteration, which supports the correct protein folding and the degradation of misfolded species through the ubiquitin-proteasome system and the autophagy-lysosomal system.^45^ In turn, misfolded proteins induce mitochondrial damage, impacting their structure, biogenesis, respiration, and fatty acid metabolism. Consequently, the production of reactive oxygen species (ROS) exacerbates protein misfolding.^11^^,^^46^ Mitochondrial oxidative capacity is usually most reduced in patients with AL compared ATTR cardiac amyloidosis, probably due to the direct interaction between AL fibrils and mitochondrial proteins.^23^ In our cohorts, AL*λ* patients showed a greater correlation between proteins involved in peroxisomal *β*-oxidation. A metabolic shift toward this pathway is a hallmark of heart failure and, although it is not related to ATP production, its role may be to compensate for reduced mitochondrial function. However, conversely, this shift triggers a cascade of energy failure, toxic lipid buildup, and oxidative stress that actively promotes the progression of heart failure.^47^

In both groups of patients considered in the study, we observed a global increase of ECM-related proteins, whose dysregulation has previously been linked to cardiac amyloidosis.^48^ Along with the complement system, the ECM-related protein abundance was higher in ATTR group, confirming previously reported findings^17^^,^^20^^,^^49^ and candidating the corresponding processes as hallmarks of the ATTR endomyocardial proteome. A bidirectional interplay between complement system and mitochondrial dysfunction has already been reported.^50^ Complement system anomalies have also been linked to thromboembolism and bleeding, which are among the most common complications of patients with ATTR.^37^^,^^38^ A further factor triggering such events could be the capillary fragility caused by the deposition of amyloid in blood vessels, while other studies reported that oligomeric ATTR fibrils directly affect endothelial cell function, impacting vascular permeability.^51^ However, abnormal bleeding has often been observed in patients with AL amyloidosis also.^52^

Previous studies reported that massive accumulation of ATTR could ultimately affect tissue architecture through mechanical stress that compromises cardiomyocyte function.^12^ The role of some proteins exclusively identified in the patients under consideration fits well into this framework. In particular, EPPK1 acts as a protective element by controlling the reorganization of keratin filaments in response to various types of stress, including mechanical stress.^30^ Similarly, ADORA3 and its signaling pathway were correlated to the cardioprotective actions to counter hemodynamic stress,^53^ and it is in well in agreement with the measures performed in our cohorts. Indeed, ADORA3, along with KIAA1958 and C1QTNF5, was found exclusively in the ATTR group, whose components showed better cardiac performance. Furthermore, whereas KIAA1958 is an uncharacterized protein with unknown function, C1QTNF5 is produced by heart muscle myocytes. Its expression increases with mitochondrial dysfunction, and under these conditions, C1QTNF5 acts as an AMPK activator, which regulates energy balance.^54^

Regarding the AL*λ* patients included in our cohorts, both quantitative and weighted network analyses converged in highlighting the role of vesicle- and microtubule-mediated transport, including endocytosis and phagocytosis. These processes and the multitude of elements , such as exosomes, microvesicles, apoptotic bodies, etc, that participate in them indicate intracellular signaling as well as the communication between the heart and other organs. They, therefore, underlie crucial cardiac mechanisms.^55^ A direct link between AL amyloidosis and vesicle-mediated transport could lie in the macropinocytosis mechanism by which AL enter cells.^56^ In cardiac fibroblasts, it was shown that AL in a fluid phase are internalized by an endosomal/lysosomal pathway and then secreted into the extracellular medium through trafficking mechanisms mediated by microtubules.^57^ Clues suggesting a potential role for microtubules in AL pathophysiology had already emerged in our previous study that aimed to clarify protein network alterations in abdominal subcutaneous adipose tissue of AL*λ* and AL*κ* patients.^19^ For instance, in the present study, the regulation of chaperonin containing tailless complex polypeptide 1 (TCP-1), also named TRiC (TCP-1 ring complex or CCT/TricC complex) was highlighted. In addition to promoting protein folding of actins and tubulins,^58^ this complex is involved in autophagic processes, and its impairment limits the degradation of misfolded aggregated proteins.^59^ Furthermore, CCT/TricC has recently been shown to be a component of extracellular vesicles (EVs) released by human Jurkat cells and mouse T lymphoblasts, thus suggesting a relationship with the endolysosomal system.^60^

In addition to EPPK1, the most potentially interesting proteins emerged from the quantitative and topological proteomic landscape included ADGRL1 and VANGL2. ADGRL1, formerly known as latrophilin-1, is involved in cell adhesion and signal transduction, and it is crucial for neuronal development and synapse function.^61^ Moreover, it has recently been described as a regulator of glucose and energy homeostasis, making it critical for cardiac physiology.^62^^,^^63^ The lack of ADGRL1 in both patient cohorts, combined with the reciprocal regulation of G-protein subunits (GNB1, GNB2, GNB3, GNB4, GNG12, and GNAI2), could be consistent with a compensatory cellular adaptation to maintain the homeostasis of cAMP,^34^ an intracellular second messenger that plays a key role in the regulation of cardiac function, including heart rate and the strength of contraction.^64^ Along with the role of ADGRL1 as regulator of synaptic processes,^65^ these observations put the spotlight on the cardiac endogenous neurotransmitter system and how these are influenced by amyloid deposition. From our results, neuronal proteotoxicity could be more correlated to ATTR, thus supporting previous reported findings.^66^ This hypothesis is supported by VANGL2 and NMDA receptor as quantitative and topological signatures of the ATTR patients present in our cohorts. In a mouse model of Alzheimer’s disease, it was found that *β*-amyloid assists VANGL2 in disassembling synapses.^67^ NMDA receptor, in turn, regulates synaptic plasticity, and its dysfunction contributes to the pathogenesis of major neurodegenerative diseases.^68^ At the cardiac level, it helps regulate blood pressure, endothelial permeability, heart rate, and contraction. Its activity can also promote ventricular arrhythmias, heart failure, and pulmonary hypertension^69^; therefore, the identification of weighted hubs or bottlenecks enriched in NMDA receptor activation may signal impairment or stress of these processes.

The pattern of results outlined by our study supports previously reported findings and provides insights into potential key players characterizing the endomyocardial proteome of AL*λ* and ATTR amyloidosis patients in our cohorts. To unravel these features, we emphasize the importance of analyzing whole biopsies rather than microdissected specimens. Although they represent complex samples, we have ascertained that they are a valuable source of qualitative and quantitative information, contributing to reconstruct the puzzle of intracellular and extracellular molecular relationships triggered by amyloid deposition.

In addition to strengthening the hypotheses on the pathogenic mechanisms of AL*λ* and ATTR, such as macropinocytosis for cellular uptake of AL and the massive extracellular accumulation of ATTR fibrils, our results suggest a number of proteins (ADGRL1, EPPK1, VANGL2, ADORA3, KIAA1958, and C1QTNF5) as candidates to be differentially abundant and to be tested in new cohorts; a representative example could be C1QTNF5, whose high level in the sera of obese/diabetic mice was proposed as a putative marker of mitochondrial dysfunction.^54^

### Limitations of the study

Although we addressed the age and gender heterogeneity of our cohorts through proper statistical correction, we are aware that our findings should be validated and further explored in new larger cohorts. In this scenario, studies that can leverage clinical, physiological, genotypic, and biometric parameters could certainly benefit, allowing *a priori* stratification that would improve correlation with molecular findings. Unfortunately, due to the limited number of participants/patients at our disposal, we were unable to pursue this strategy in our study. In addition to the aforementioned age and gender gap, a further weakness could be hidden in the control group including participants with diverse clinical history. However, their fitness was partly validated by the homogeneity of their profiles, and the low number of proteins differentially abundant by gender and age. Finally, it must be highlighted that while whole tissue proteomics analysis allows us to globally depict the effect of amyloid deposition on surrounding tissue, it makes it difficult to attribute such effects to specific cell types.

These limitations should be considered while interpreting our results, but they do not detract from the value of the molecular insights gained from these precious samples. Indeed, they represent a useful source of information that could inspire further investigations on cardiac amyloidosis.

## Resource availability

### Lead contact

Further information and requests for resources and reagents should be directed to and will be fulfilled by the lead contact, Dario Di Silvestre (dario.disilvestre@itb.cnr.it).

### Materials availability

This study did not generate new unique reagents.

### Data and code availability

• The MS data have been deposited to the ProteomeXchange consortium via the PRIDE partner repository with the dataset identifier PRIDE: PXD033168.
• This paper does not report original code.
• Any additional information required to reanalyze the data reported in this paper is available from the lead contact upon request.

## Acknowledgments

The authors acknowledge the patients, clinicians, and laboratory staff at the Sahlgrenska University Hospital and at the University of Gothenburg, who provided us with the primary datasets enabling this study. This work was funded by Swedish governmental funding of Clinical Research (ALFGBG_1006161) and Italian Ministry of University and Research (PRIN2022: 2022Z2TE5P, PRIN2022: 2022LNHZAP, and PRIN 2022: 2022YA9C33). LC-MS/MS was performed at the Proteomics Core Facility, Sahlgrenska academy, Gothenburg University, with financial support from SciLifeLab and BioMS. The authors also thank all members of the lab for their support.

## Author contributions

R.V., methodology and original draft writing; A.L., methodology and original draft writing; F.N., methodology and manuscript revision; J.N., methodology and manuscript revision; S.H., methodology; A.O., methodology and manuscript revision; K.K., methodology and manuscript revision; K.V., methodology; J.S., methodology; E.B., methodology; G.L., methodology and manuscript revision; P.M., methodology and manuscript revision; F.B., methodology and manuscript revision; and D.D.S., conceptualization, supervision, and writing – review and editing.

## Declaration of interests

The authors declare no competing interests.

## STAR★Methods

### Key resources table

**Table:** 

REAGENT or RESOURCE	SOURCE	IDENTIFIER
**Chemicals, peptides, and recombinant proteins**		

Congo Red solution	CR 3 g/L, NaCl 3 g/L, 80% ethanol, 0.01% NaOH	In-house
99mTc-Hydroxymethylene diphosphonate (HDP)	Cappelli et al.^70^	https://doi.org/10.1007/s12350-017-0922-z
99mTc-3,3-diphosphono-1,2-propanodicarboxylic acid (DPD)	Cappelli et al.^70^	https://doi.org/10.1007/s12350-017-0922-z
Hematoxilin	Mayer’s hemalum	Merck, 109249
Eosin	Mayer’s hemalum	Merck, 109249

**Deposited data**		

Raw proteomics spectra	https://www.ebi.ac.uk/pride/	PRIDE: PXD033168

**Software and algorithms**		

Proteome Discoverer v2.5	ThermoFisher Scientific	NA
Limma R Package	https://bioconductor.org/packages/release/bioc/html/limma.html	https://doi.org/10.1093/nar/gkv007
R v4.4.2	https://www.r-project.org	NA
JMP v15.2	SAS Institute	NA
STRING Database v12.0	https://string-db.org/	https://doi.org/10.1093/nar/gkaa1074.
ggtree	https://www.bioconductor.org/packages/release/bioc/html/ggtree.html	https://doi.org/10.1111/2041-210X.12628
ggplot2	https://ggplot2.tidyverse.org	https://doi.org/10.1007/978-0-387-98141-3
Circos	https://circos.ca/software/download/circos/	https://doi.org/10.1101/gr.092759.109
Human Protein Atlas v23	https://v23.proteinatlas.org/	https://doi.org/10.1126/science.1260419
Cytoscape v3.10.3	https://cytoscape.org/download.html	https://doi.org/10.1101/gr.1239303
igraph	https://igraph.org	https://doi.org/10.5281/zenodo.7682609
COPPIs algorithm	https://github.com/lomi95/CoPPIs	https://doi.org/10.1093/bib/bbaf146
α-value algorithm	Brambilla et al.^71^	https://doi.org/10.1182/blood-2011-07-365510
KEGG PATHWAY Database	https://www.genome.jp/kegg/pathway.html	https://doi.org/10.1093/nar/28.1.27
Reactome Pathway Database	https://reactome.org/	https://doi.org/10.1093/nar/gkad1025
WikiPathways	https://www.wikipathways.org/	https://doi.org/10.1093/nar/gkad960
BINGO 2.44	https://apps.cytoscape.org/apps/bingo	https://doi.org/10.1093/bioinformatics/bti551
Centiscape	https://apps.cytoscape.org/apps/centiscape	https://doi.org/10.1093/bioinformatics/btp517

### Experimental model and study participant details

The raw mass spectra concerning the subjects considered for this study were previously published in the context of cardiac amyloidosis subtyping by mass spectrometry-based proteomics of endomyocardial biopsies; a detailed description of sample preparation and LC-MS/MS analysis is given in Noborn et al.^21^ The original set of data contains the profiles of 12 controls and 75 patients belonging to four groups, such as AL*λ*, ATTR, AA, and AL*κ*; these patients received a histopathological diagnosis of cardiac amyloidosis based on biobank-stored endomyocardial biopsies obtained consecutively between 1995 and 2020 as part of their heart failure evaluation. The study was approved by the Research Ethics Board at the Sahlgrenska Academy, University of Gothenburg, Sweden, following the Helsinki Declaration (approval number: 286-18).

For the purposes of this study, patient selection criteria primarily included the Congo red score, and MS-based diagnostic confidence (≥70%) calculated through a modified version of the *α*-value algorithm.^21^^,^^71^ Patients whose clinical classification was discordant with the MS-based one were excluded. In the same way, outlier protein profiles reporting a total protein count far from the mean of the entire group were not considered. Moreover, due to the low number of patients, the AA and AL*κ* groups were not considered.

Based on these premises, we selected the data corresponding to 12 control cases (C) stemming from unused donor heart explants, 19 patients affected by AL lambda (AL*λ*), and 20 patients affected by ATTR amyloidosis (ATTR). Specifically, we arranged a first cohort including an equal number of control subjects and patients affected by AL*λ* and ATTR amyloidosis (*n* = 12 per group), while a second cohort was arranged to highlight the differences between patients affected by AL*λ* (*n* = 19) and ATTR (*n* = 20) amyloidosis; a comparable sample size aims to obtain a more reliable and statistically robust label-free quantification, and it is an even more fundamental detail for the reconstruction and comparison of co-expression networks whose require a certain sample size (≥20).^22^^,^^72^

Control samples were selected to be non-amyloidogenic heart tissues, and were taken from the same areas as those used for patient samples. They were collected from hearts of 5 men and 7 women, mean age 59 years (range 31–75 years), who for various reasons had not been considered suitable for transplantation. The samples came from the left ventricle (free wall 8, septum 4). They were rapidly frozen in liquid nitrogen and stored at −80°C. Protein extraction was performed from 10 *μ*m-thick cryostat sections. Separate sections were stained with hematoxylin and eosin (H&E) to verify representativeness and normal histology and with Congo red to exclude the presence of amyloid.

ATTR patients selected for the first cohort were all affected by non-hereditary ATTR amyloidosis (ATTRwt), while in the second cohort only 2 out of 20 patients were affected by hereditary ATTR amyloidosis (ATTRv). At the time of diagnosis, patients were receiving only guideline-directed medical therapy (GDMT) for heart failure.

Further details including age, gender, biometric data, clinical parameters and hemodynamic measurements are available in Table S1.

### Method details

#### Congo red staining

All investigated patients had cardiac amyloidosis verified by Congo red staining of endomyocardial formalin fixed and paraffin embedded specimens. The proteomic investigations were performed on a separate fresh frozen biopsy specimen. To better visualize the Congo red positive deposition the specimens were scanned with fluorescence microscopy using filters for Texas Red (Hamamatsu S60). Semiquantitative estimation was performed by applying four grades: 0, no amyloid was detected; +, small foci of amyloid were detected (≤5%); ++, widespread amyloid deposition affecting 5–20% of the tissue; +++, massive amyloid deposition replacing ≥20% of the myocardium.

#### Biometric data, clinical parameters and hemodynamic measurements

Clinical course, laboratory tests, echocardiography, endomyocardial biopsy, cardiac magnetic resonance and scintigraphy data were collected as reported in Noborn et al. 2022.^21^ For all patients, we report here height, weight, body mass Iindex (BMI), creatinine level, N-terminal prohormone fragment of B-type natriuretic Ppeptide (NT-proBNP), left ventricular ejection fraction (LVEF), left ventricular end-diastolic diameter (LVEDd), right atrial pressure (RAp), right ventricular systolic pressure (RVSp), right ventricular end-diastolic pressure (RVEDp), pulmonary artery systolic pressure (PASp), pulmonary artery diastolic pressure (PADp), pulmonary artery mean pressure (PAMp), pulmonary capillary wedge pressure (PCWp), cardiac output (CO), mixed venous oxygen saturation (SVO2). Specifically, the scintigraphy analyses were performed either with 99mTc-Hydroxymethylene diphosphonate (HDP) or 99mTc-3,3-diphosphono-1,2-propanodicarboxylic acid (DPD).^70^

#### Raw mass spectra processing

The raw mass spectra concerning the subject considered for this study were reprocessed by means of the Proteome Discoverer 2.5 software (Thermo Scientific) using the Processing and Consensus workflows (Figure S7). The *Homo sapiens* proteome, including isoforms, was downloaded from UniProt (www.uniprot.org) in November 2024 and it was set as reference database. Trypsin was set as a digestion enzyme (with a maximum of 3 missed cleavages allowed), while methylthiolation on Cys residues was set as a dynamic modification. Precursor mass tolerance and fragment mass tolerance were set at 10 ppm and 0.05 Da respectively. The Consensus Workflow was built according to ThermoFisher Scientific default workflow for DDA protein identification and annotation. Percolator node was used with a target-decoy strategy to give a final false discovery rate (FDR) ≤ 0.01 based on q-values, considering maximum deltaCN of 0.05. Only peptides with minimum peptide length of four amino acids, confidence at “Medium” level and rank 1 were considered and counted only for the top-scored protein; peptides without a protein reference were removed. Protein grouping and strict parsimony principle were applied, and the PSM Grouper used a site localization probability threshold ≥75%. The resulting protein lists were exported in excel files reporting the Peptide Spectrum Matches (PSMs) for each identified protein. Finally, the number of proteins and PSMs per group was compared through Kruskal-Wallis and Dumm test.

#### Enrichment analysis

A first functional assessment of the characterized protein profiles was performed using the functional annotation tool inserted in the STRING database.^73^ The entire profile of each subject was processed and the enriched COMPARTMENTS, GO Biological Process (BP), GO Molecular Function (MF), GO Cellular Component (CC), KEGG, Reactome and WikiPathways terms were extracted (FDR ≤0.05). The enrichment profiles were then aligned and compared by linear discriminant analysis (LDA); those with F ratio ≥3.5 and *p*-value ≤0.05 were selected as differentially enriched among control, AL*λ* and ATTR subjects. Finally, they were represented by tree charts built using the ggtree and ggplot2 R packages^74^ and the Circos tool.^75^

#### Protein atlas data evaluation

Homo sapiens RNA immune cell data were retrieved from the Human Protein Atlas v.23.^76^ Proteins specifically found in at least 5 AL*λ* patients (and never in ATTR), or in at least 5 ATTR patients (and never in AL*λ*) were extracted and the expression values across immune cell types were retrieved. A bar plot was generated by summing the number of transcripts per million (nTPM) across immune cell types per condition.

#### Reconstruction of PPI network model and functional modules identification

A PPI network model was reconstructed using STRING Cytoscape app^73^ starting from DAPs (*n* = 422, P≤0.00001) selected by comparing C (*n* = 12), AL*λ* (*n* = 12) and ATTR (*n* = 12). Only protein-protein interactions annotated as “databases” and/or ”experiments” annotated, and with an STRING score ≥0.3 and ≥0.15 respectively, were retained. Proteins were grouped in PPI functional modules using the STRING Cytoscape app^73^ and BINGO 2.44^77^; as for BINGO 2.44, *Homo sapiens* organism, hypergeometric test and Benjamini–Hochberg FDR correction (≤0.01) were set.

#### Topological analysis of PPI and Co-expression network models

A PPI network model per group was reconstructed starting from the total proteins found in C (*n* = 12 subjects and 5,913 proteins), AL*λ* (*n* = 12 subjects and 7,072 proteins) and ATTR (*n* = 12 subjects and 6,262 proteins); also in this case, only protein-protein interactions annotated as “databases” and/or “experiments” annotated, and with a Score ≥0.3 and ≥0.15 respectively, were retained. The reconstructed models were analyzed at the topological level using Centiscape Cytoscape app,^78^ as previously reported.^79^ Diameter, Average Distance, Degree, Betweenness, Centroid, Stress, EigenVector, Bridging, Eccentricity, Closeness, Radiality and Edge centralities were calculated. Betweenness coupled with Centroid and Betweenness coupled with Bridging were used to select hubs and bottlenecks, respectively; node with both values above the average were retained.^80^ Statistical significance of topological results was tested using randomized network models^81^; *n* = 1000 random models per group were reconstructed and analyzed by *in-house* R scripts based on *igraph* (to build random models and to compute centralities), and *ggplot2* (to plot results) libraries.

#### CoPPIs algorithm

AL*λ* (*n* = 19) and ATTR (*n* = 20) protein profiles were processed by the CoPPIs algorithm to extract differentially correlated PPI functional modules (CoPPIs score ≥|2|, edge percentage ≥0.3, FDR ≤0.001) between AL*λ* and ATTR subjects.^22^ Additionally, the Spearman’s correlation score calculated from the CoPPIs, corrected for FDR and transformed, was used to weight the PPI profiles of the AL*λ* and ATTR groups. Betweenness, bridging and centroid centrality were computed on the weighted networks. Hubs and bottlenecks were extracted considering the proteins whose centrality values were above the 75th quantile in AL*λ* (or ATTR) but not in the 75th quantile of ATTR (or AL*λ*).

### Quantification and statistical analysis

The protein profiles characterized by LC-MS/MS analysis were semi-quantitatively compared using a label-free approach based on PSMs and the limma R package.^82^ Linear models were fitted to log2-transformed data, including age and gender as covariates. Conversely, to assess whether and to what extent age and gender influence protein abundance within each group, a multivariate analysis of variance (MANOVA) was used considering a *p* ≤ 0.05 after false discovery rate (FDR) correction. Prior to limma and MANOVA, the PSM values were normalized using a total signal normalization method.^80^ The limma framework was applied to both Cohort 1 (Control, *n* = 12; AL*λ*, *n* = 12; ATTR, *n* = 12) and Cohort 2 (AL*λ*, *n* = 19; ATTR, *n* = 20). In addition, pairwise comparisons (AL*λ* vs*.* Control; ATTR vs*.* Control; ATTR vs*.* AL*λ*) were performed and a volcano plot per comparison was built by plotting log-transformed fold change (*x*-axis) against the negative log-transformed *p*-value (emphy-axis). Selected DAPs (adj.P.Val ≤0.05) were further processed by principal component analysis (PCA) and hierarchical clustering. All data processing were performed using R and JMP 15.2 SAS software.

## Supplemental information

Document S1. Figures S1–S7

Table S1. Demographic, anthropometric, laboratory, and invasive hemodynamic measures

Table S2. Proteins identified in cardiac biopsies included in cohort1 (control, ALλ, and ATTR; *n* = 12 per group) and cohort2 (ALλ, *n* = 19; ATTR, *n* = 20)

Table S3. Quantitative analysis by grouping control participants and patients by gender and age

Table S4. Differentially abundant proteins extracted from cohort1 (control, *n* = 12; ALλ *n* = 12; and ATTR *n* = 12) and cohort2 (ALλ, *n* = 19; ATTR, *n* = 20)

Table S5. Differentially enriched GO terms and pathways among control (*n* = 12), and ALλ (*n* = 12) and ATTR patients (*n* = 12)

Table S6. Differentially enriched GO terms and pathways between ALλ (*n* = 19) and ATTR patients (*n* = 20)

Table S7. Unweighted hubs/bottlenecks from cohort1 and weighted hubs/bottlenecks from cohort2

Table S8. Differentially correlated GO terms and pathways between ALλ (*n* = 19) and ATTR patients (*n* = 20)
