# Supplementary material for: A journey through the molecular networks of endomyocardial biopsies to explore ATTR and ALλ cardiac amyloidosis
Source: iScience. 2026 Jul 3;29(7):116573. doi: 10.1016/j.isci.2026.116573 (PMC13355674; doi:10.1016/j.isci.2026.116573)
Supplement: Document S1. Figures S1–S7 [file mmc1.pdf]

iScience, Volume *29*

Supplemental information

A journey through the molecular networks of endomyocardial biopsies to explore ATTR and ALλ cardiac amyloidosis

Raffaello Viganò, Andrea Lomagno, Fredrik Noborn, Jonas Nilsson, Saleh Hamed, Anders Oldfors, Kristjan Karason, Kristina Vukusic, Joakim Sandstedt, Emanuele Bobbio, Göran Larson, Pierluigi Mauri, Francesca Brambilla, and Dario Di Silvestre

SUPPLEMENTARY FIGURES

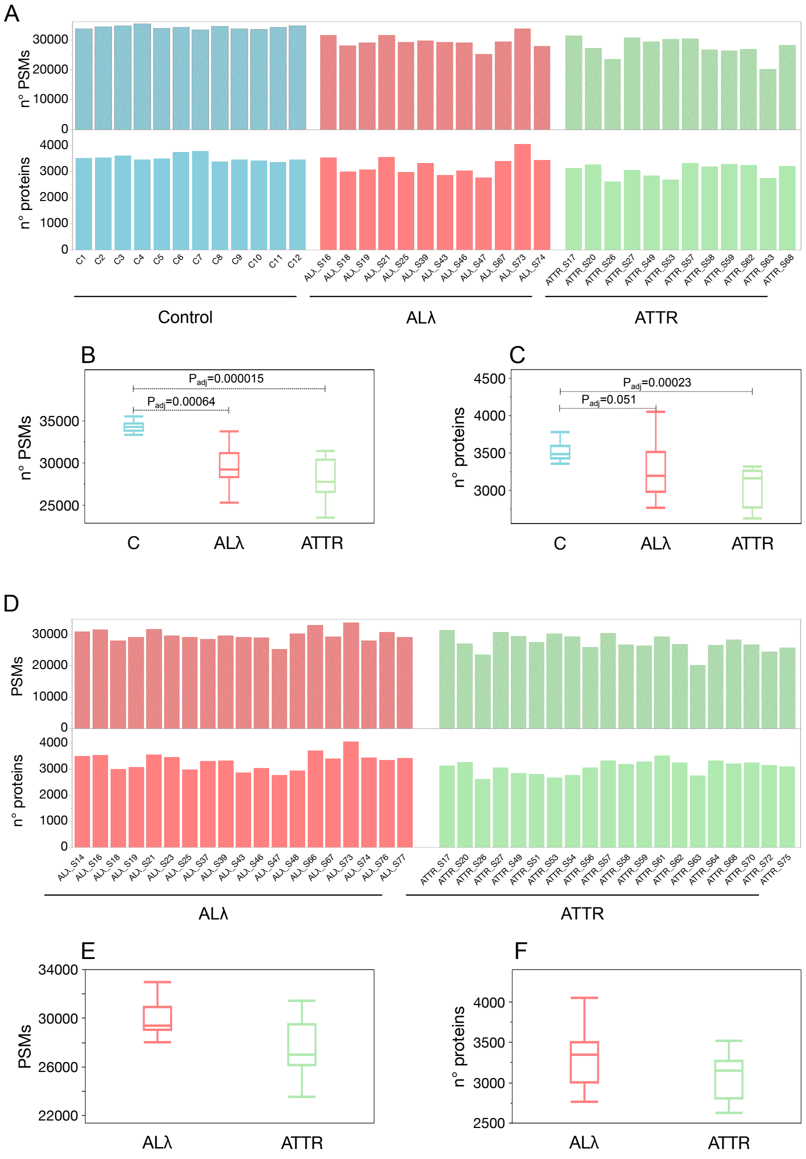

G

	Intragroup correlation [1]			Intergroup correlation[2]		
Group (n=12)	C	ALλ	ATTR	C *vs* ALλ	C *vs* ATTR	ATTR *vs* ALλ
Spearman's correlation	0,933	0,865	0, 842	0,887	0,888	0, 970
Group (n∼20)		ALλ	ATTR			ATTR *vs* ALλ
Spearman's correlation		0,871	0, 853			0, 980

[1]Average Spearman's correlation comparing all subjects belonging to the same group.

[2]Average Spearman's correlation comparing all subjects in the first group against all subjects in the second group.

Figure S1: **Protein profiling**.

Bar charts (A) and boxplots (B-C) of peptide spectrum matches (PSMs) and proteins identified in the first cohorts (C, ALλ and ATTR, n=12 per group); data are represented as a combination of mean, quantiles (25%-75%), and outliers.

Bar charts (D) and boxplots (E-F) of peptide spectrum matches (PSMs) and proteins identified in the second cohorts (ALλ, n=19: ATTR, n=20. Kruskal-Wallis and Dumm test were applied ($P \leq 0.05$); data are represented as a combination of mean, quantiles (25%-75%), and outliers.

G) Intra-group and inter-group Spearman's correlation among the characterized protein profiles. All samples allowed the identification of a comparable number of proteins and peptides, indicating homogeneous sampling. On average, a lower number of PSMs and protein per sample was identified in both ALλ and ATTR. In this scenario, ALλ showed a greater variance that could suggest a more heterogeneous set of protein profiles. However, all groups, and especially the control one, showed a high intra-group correlation score.

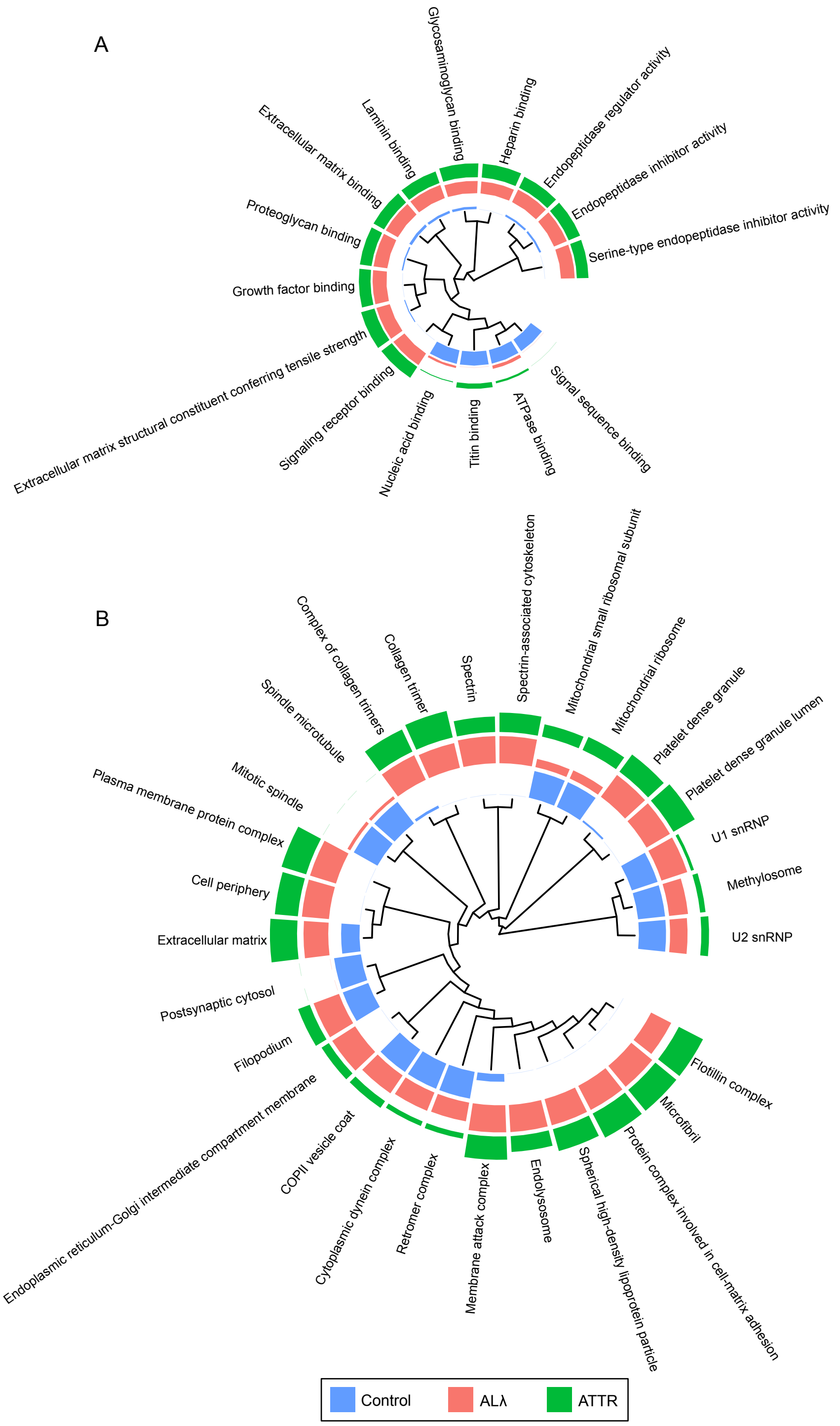

Figure S2: **Functional enrichment**.
A) Molecular Functions (MFs) and B) cellular components (CCs) differentially enriched by comparing the protein profiles of the first cohort (C, ALλ and ATTR, n=12 per group). Bars in Circos graphs indicate the normalized number of proteins found per GO term. The hierarchical clustering tree groups GO terms based on the number of protein they share. All CCs and MFs were enriched with FDR$\leq$0.05, while those differentially enriched were extracted by Linear Discriminant Analysis (LDA) (P$\leq$0.01) and DAve index $\geq|0.4|$ (**see Table S5**).

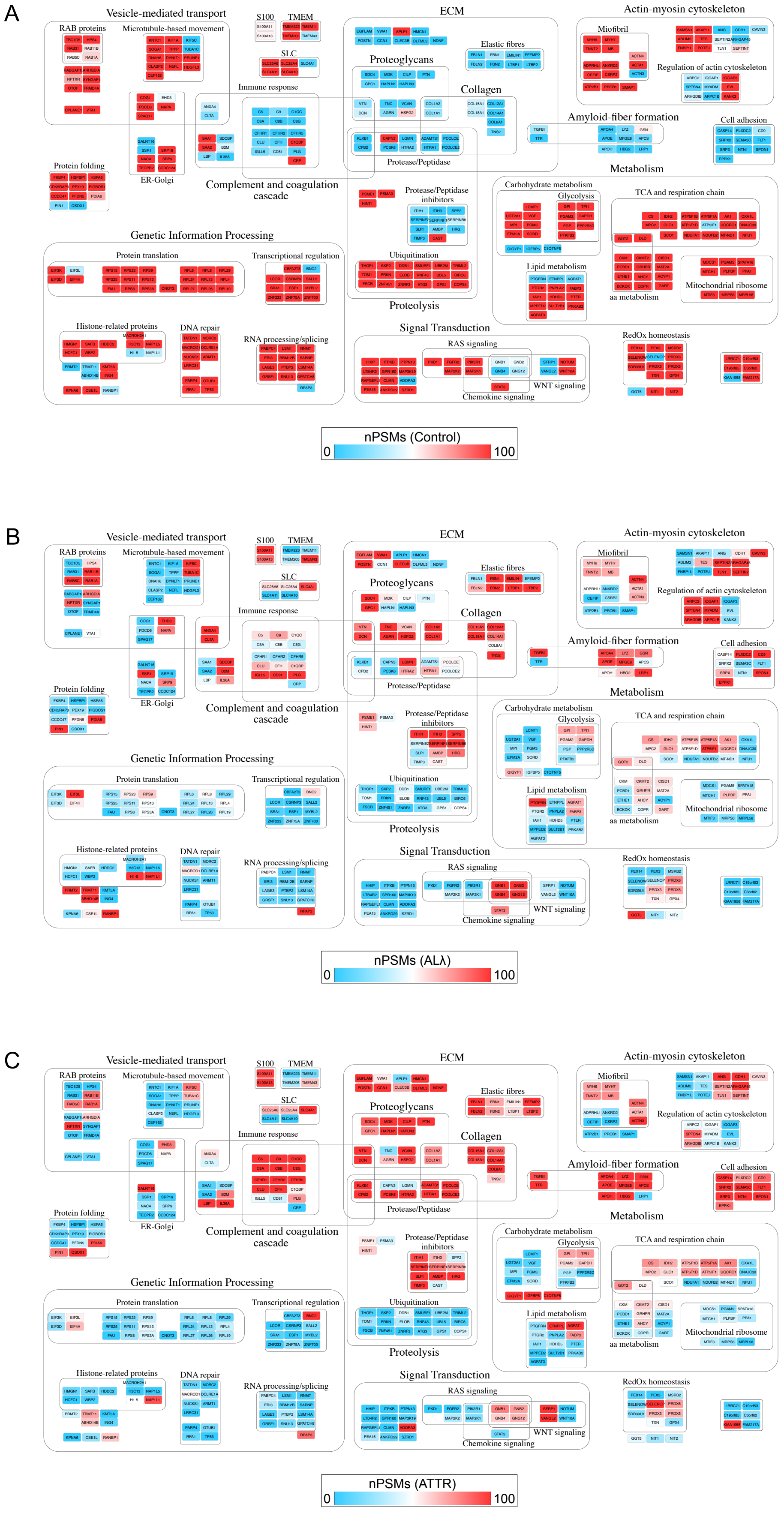

Figure S3: **Network analysis.**
Protein-Protein Interaction (PPI) functional modules and abundance level in A) control, B) ALλ and C) ATTR cardiac biopsies (First cohort, n=12 per group). The network model was reconstructed starting from 422 differentially abundant proteins (DAPs) extracted by comparing C vs ALλ (n=305, adj.P.Val $\leq$0.00001) and C vs ATTR (n=341, adj.P.Val $\leq$0.00001); only STRING database (score$\geq$0.3) and experiments (score$\geq$0.15) annotated interactions were considered. Functional modules were defined using STRING Cytoscape apps (P$\leq$0.05). The color code, from blue to red, is based on the normalized average PSM values (range 0-100); it indicates proteins with low (blue) and high (red) relative abundance.

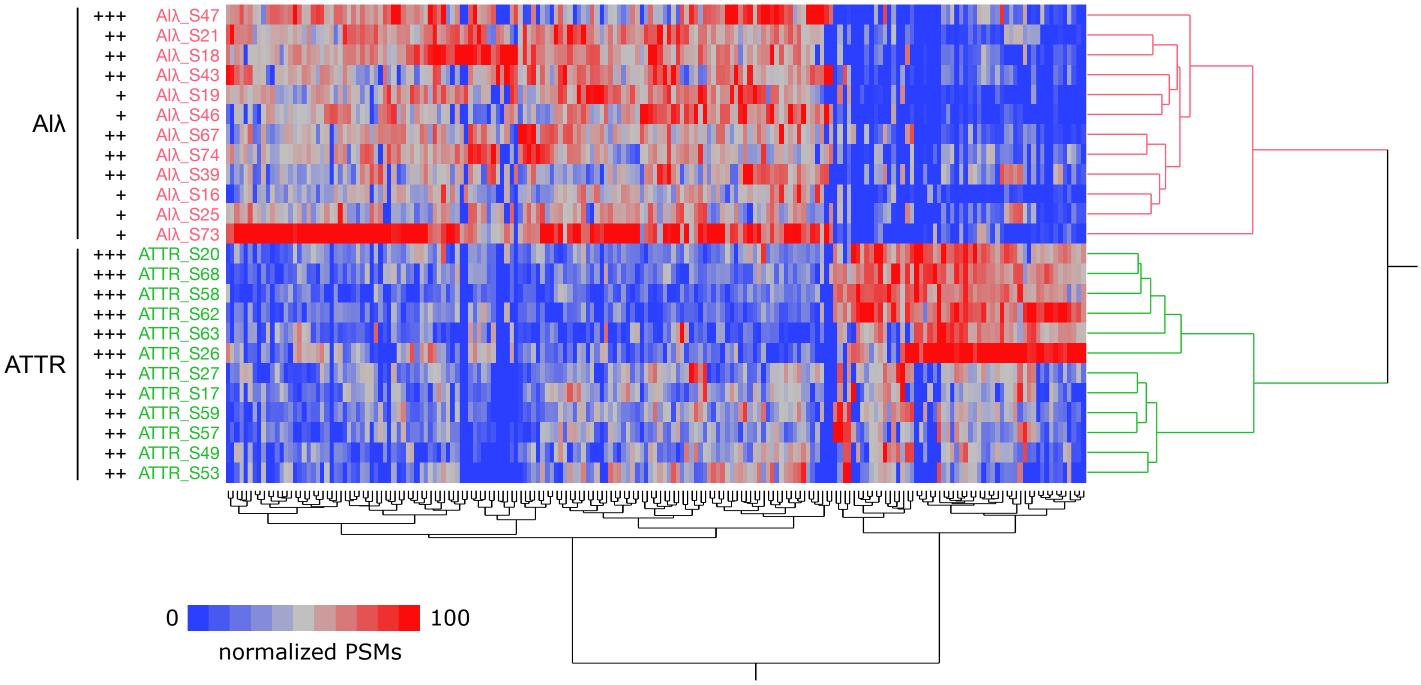

Figure S4: **Hierarchical clustering**.
It shows differentially abundant proteins (DAPs, n=191, adj.P.Val $\leq$0.05) extracted by comparing ALλ and ATTR protein profiles included in the Cohort 1 (n=12 per group). Heatmap distinguished two subgroups of ATTR patients that showed a good correlation with the corresponding Congo red staining score (+, ++, +++).

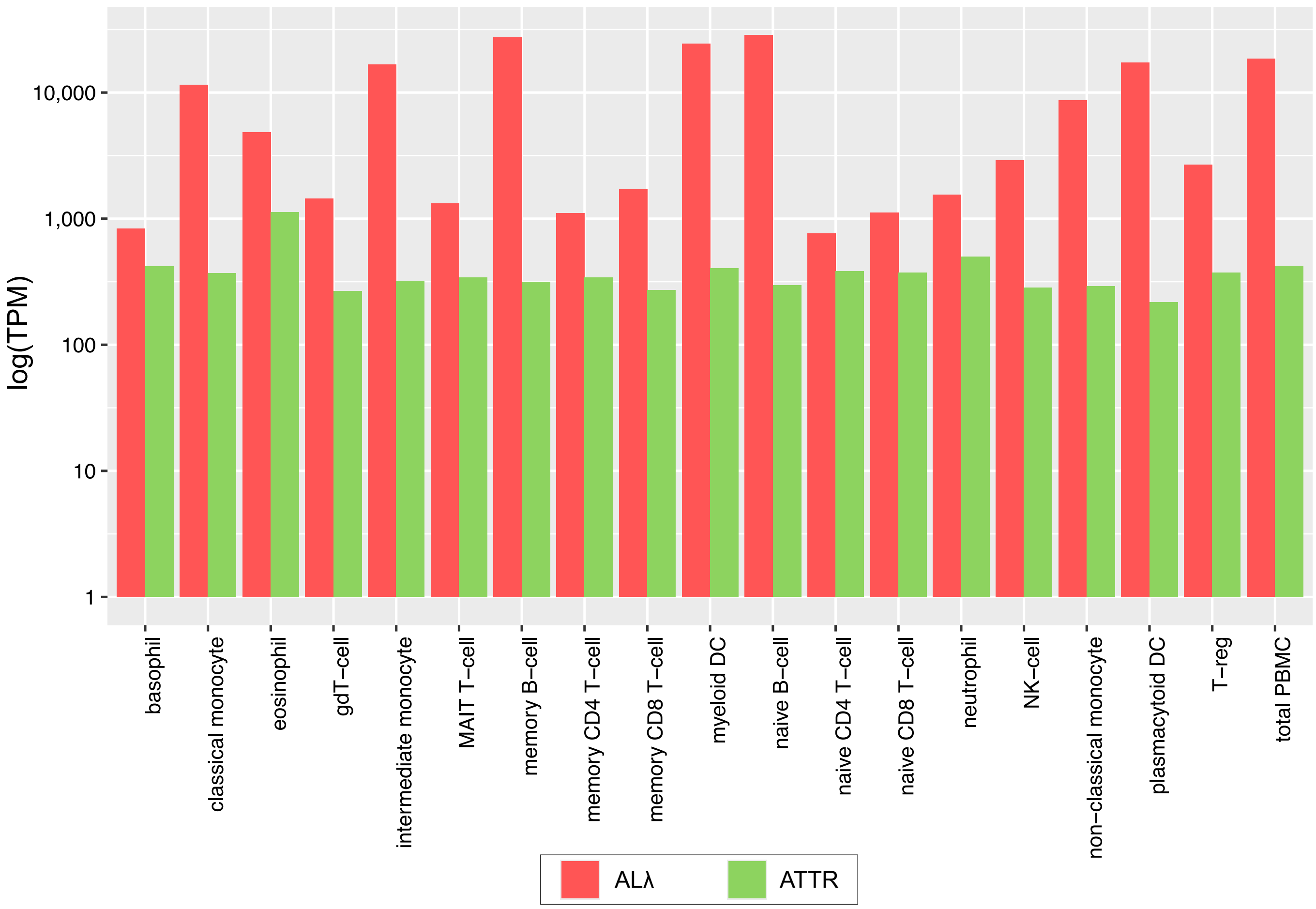

Figure S5: **Immune cells RNA expression**.
The bar graph shows RNA expression in *Homo sapiens* immune cells of proteins found in at least 5 ALλ patients (and never in ATTR), or in at least 5 ATTR patients (and never in ALλ); specifically, 40 and 53 proteins were considered for ALλ and ATTR, respectively. Bars were generated by summing the number of transcripts per million, as log(TPM), across immune cell types per condition; data were retrieved from the Human Protein Atlas v.23.

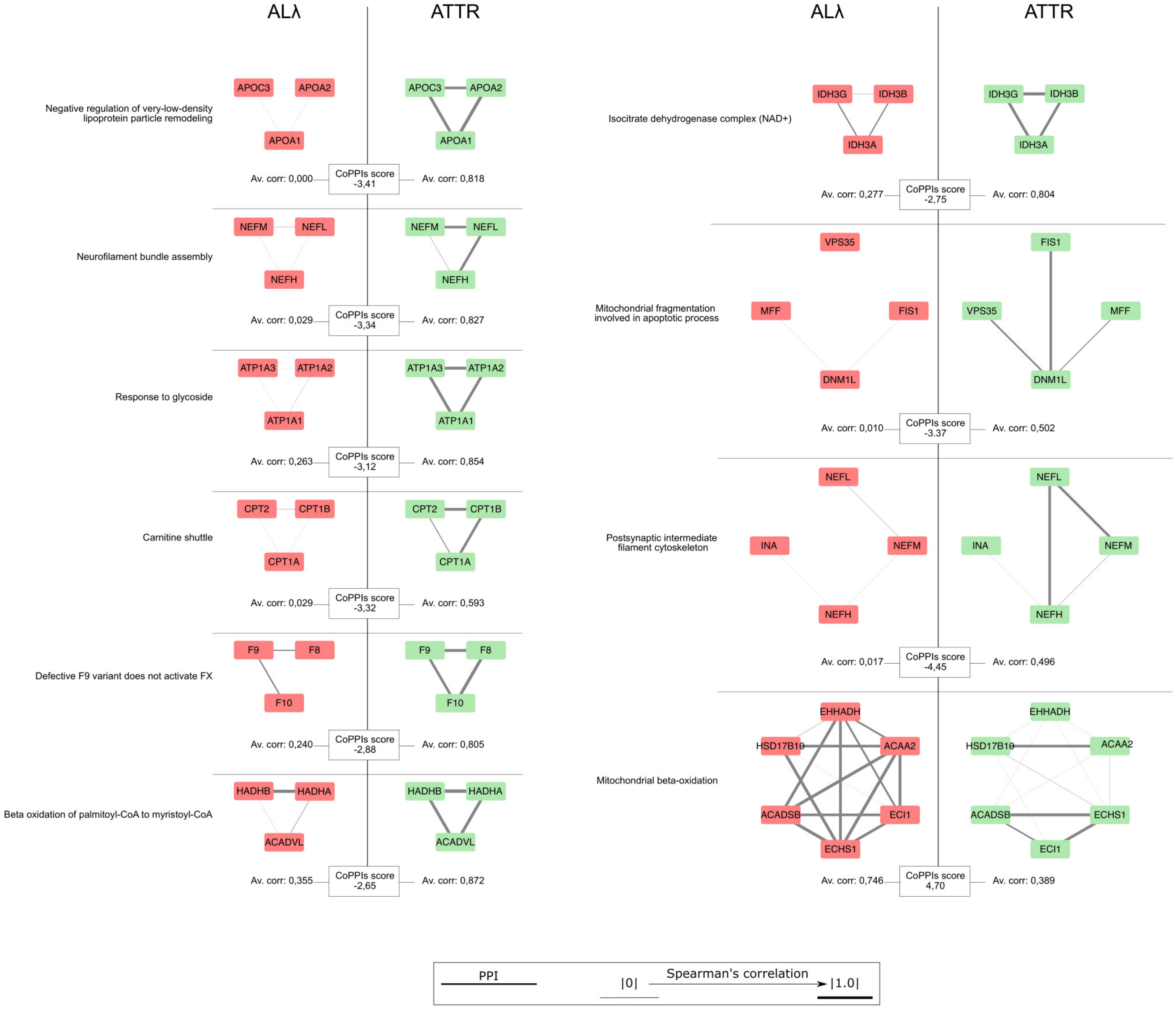

Figure S6: **Differentially correlated PPI functional modules by comparing ALλ and ATTR protein profiles**.
ALλ (n=19) and ATTR (n=20) protein profiles were processed by the CoPPIs algorithm. Differentially correlated PPI functional modules were selected by considering CoPPIs score$\geq |2|$, edge percentage coverage $\geq$30%, and FDR$\leq$0.001). Only database (STRING score$\geq$0.3) and experiments (STRING score$\geq$0.15) annotated PPIs were considered. Edges were weighted through the *Spearman*'s score transformed by CoPPIs. Specifically, a positive CoPPIs score indicates PPI modules most correlated in ALλ, whereas a negative CoPPIs score indicates PPI modules most correlated in ATTR

A B

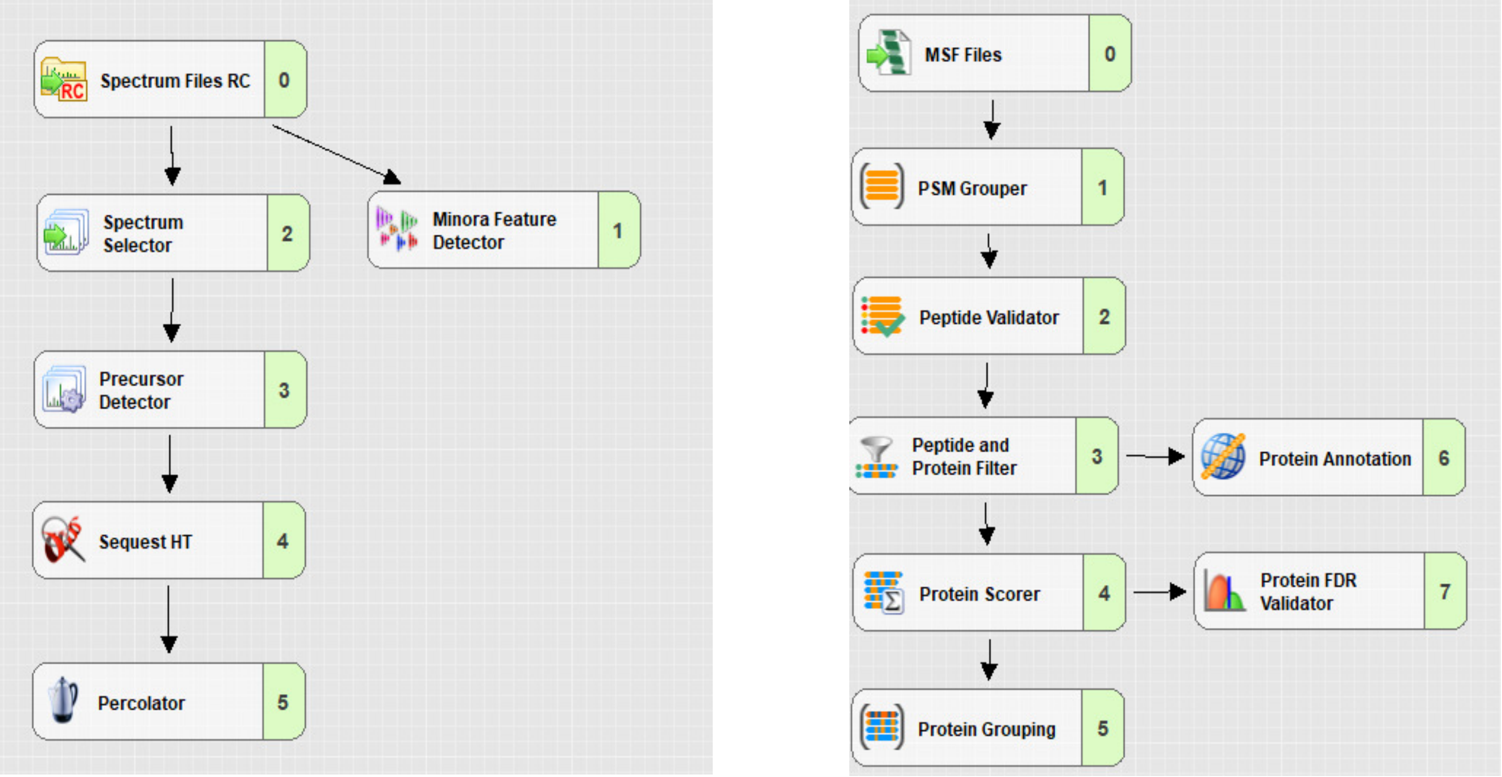

Figure S7: **Proteome Discoverer 2.5 workflows**.
Processing and Consensus workflows adopted for processing the proteomic raw data obtained by analyzing the endomyocardial biopsies from controls, and patients affected by ATTR and ALλ amyloidosis.
A) The Processing workflow included the following nodes: Spectrum Files RC (0), Spectrum Selector (2), Minora Feature Detector (1), Precursor Detector (3), Sequest HT (4) as a search engine, and Percolator (5) for results validation.
B) The Consensus workflow was composed as follows: MSF Files (0); PSM Grouper (1); Peptide Validator (2); Peptide and Protein Filter (3); Protein Annotation (6); Protein Scorer (4); Protein FDR Validator (7) and Protein Grouping (8).
